# Combined Hepatocholangiocarcinoma Associated with Humoral Hypercalcemia of Malignancy and Chronic Inflammatory Demyelinating Polyneuropathy

**DOI:** 10.1155/2019/3418950

**Published:** 2019-06-24

**Authors:** Ruben Manuel Luciano Colunga Biancatelli, Marco Ciacciarelli, Alessandro Polidoro, Piera Clemenzi, Viviana Congedo, Leonardo Calvosa, Eleonora D'Armiento, Carmen Misurale, Davide Bellini, Stefano Badia, Massimiliano Mancini, Vincenzo Petrozza, Luigi Iuliano

**Affiliations:** ^1^Department of Medico-Surgical Sciences and Biotechnologies, Internal Medicine Unit, ICOT Hospital, “Sapienza” University of Rome, Via Franco Faggiana 34, 04100 Latina, Italy; ^2^Department of Radiological Sciences, Oncology and Pathology, ICOT Hospital, “Sapienza” University of Rome, Via Franco Faggiana 34, 04100 Latina, Italy; ^3^Department of Medico-Surgical Sciences and Biotechnologies, Pathology Unit, ICOT Hospital, “Sapienza” University of Rome, Via Franco Faggiana 34, 04100 Latina, Italy

## Abstract

Paraneoplastic syndromes are often a diagnostic challenge to doctors and may have a heterogeneous presentation, including humoral hypercalcemia of malignancy (HHM), most commonly caused by squamous cell cancer and renal, ovarian, endometrial, and breast cancer. Chronic inflammatory demyelinating polyneuropathy (CIDP) has been described in patients affected by several types of cancer, especially hematologic malignancies, and a possible paraneoplastic pathogenesis of this neurological disease has been suggested. This report describes a 56-year-old man with a history of CIDP diagnosed 3 months earlier and persistently elevated aminotransferases for 18 months who was admitted to our internal medicine unit with abdominal pain, fatigue, and severe hypercalcemia with low serum intact parathyroid hormone. Parathyroid hormone-related protein (PTH-rP) was markedly high. Liver imaging showed a large hepatic mass in the right lobe, and percutaneous ultrasound-guided biopsy revealed histopathological findings consistent with a combined hepatocholangiocarcinoma (CHCC). We supposed that both HHM and CIDP could represent a paraneoplastic manifestation of CHCC.

## 1. Introduction

Paraneoplastic syndromes are often a diagnostic challenge to doctors and may present as any of a wide variety of clinical syndromes resulting from the release of self-peptides or self-like peptides to the production of autoimmune antibodies. Humoral hypercalcemia of malignancy (HHM), hypercalcemia caused by systemic secretion of parathyroid hormone-related protein (PTH-rP) by malignant cells, is considered a paraneoplastic syndrome, and it is most commonly caused by squamous cell cancer (e.g., of the head and neck, esophagus, cervix, and lung) and renal, ovarian, endometrial, and breast cancer [1]. Paraneoplastic neurological syndromes (PNS) may have a heterogeneous presentation, including polyneuropathies, and can occur with any type of cancer, especially small cell lung cancer, ovarian and breast cancer, neuroendocrine tumors, thymoma, and lymphoma. Interestingly, PNS more commonly develop prior to the cancer diagnosis and are frequently associated with antineuronal antibodies that can be measured in serum and cerebrospinal fluid [2]. Chronic inflammatory demyelinating polyneuropathy (CIDP) is an autoimmune peripheral neuropathy frequently described in association with several types of cancer, especially hematologic malignancies. We present the first case of combined hepatocholangiocarcinoma (CHCC) presenting with HHM in a patient with a CIDP.

## 2. Case

A 56-year-old man was referred to our internal medicine unit with abdominal pain, fatigue, and persistently elevated aminotransferases for 18 months. Three months earlier, he had been evaluated for numbness and weakness starting over the distal aspects of his four limbs and slowly progressing proximally over the last three years. Neurological examination performed at that time revealed walking difficulties and moderate muscle weakness in both lower and upper limbs (*F* = 3.5-4), generalized tendon areflexia, and mild sensory loss with stock and glove distribution. Electromyography/electroneurography (EMG/ENG) showed diffusely reduced motor and sensory nerve conduction velocity (mean motor nerve conduction velocity 22 m/s), with a dishomogeneous pattern, and absent *F* waves. Isolated hyperproteinorrachia (1.15 g/L) was found on cerebrospinal fluid (CSF) examination. The clinical, EMG/ENG, and CSF results were consistent with a diagnosis of CIDP, which was subsequently successfully treated with IV immunoglobulins.

On admission, the patient's temperature was 36°C, heart rate 125 beats per minute, blood pressure 110/80 mmHg, and oxygen saturation 94% while he was breathing ambient air. On physical examination, peripheral edema, bibasal pulmonary rales, hepatomegaly, and severe sensory and motor deficits located to the upper and lower limbs were noted. Laboratory data on admission showed the following values: aspartate aminotransferase (AST) 166 IU/L (normal range 17-59 IU/L), alanine aminotransferase (ALT) 64 IU/L (normal range 21-72 IU/L), gamma glutamyl-transferase 358 IU/L (normal range 15-73 IU/L), alkaline phosphatase 173 IU/L (normal range 38-126 IU/L), total bilirubin 1.28 mg/dL (normal range 0.20-1.30 mg/dL), albumin 2.8 g/dL (normal range 3.6-5.5 g/dL), lactate dehydrogenase 993 IU/L (normal range 313-618 IU/L), total serum calcium level 14.1 mg/dL (normal range 8.8-10.2 mg/dL), phosphorous 2.8 mg/dL (normal range 2.9-4.8 mg/dL), C-reactive protein 14.6 mg/dL (normal range < 1.0 mg/dL), and ferritin 1669 ng/mL (normal range 20-325 ng/mL). Viral markers for hepatitis B and C were negative. Serum intact PTH was low (4 pg/mL) (normal range 20-104 pg/mL). Moreover, anti-ganglioside antibodies (GD1bIgG and GM1IgG) were found in serum. Given the predominantly infiltrative pattern of altered liver function tests and the severe hypercalcemia with low serum intact PTH, imaging studies and further blood tests were performed to rule out cancer or metastases.

**Figure 1:**
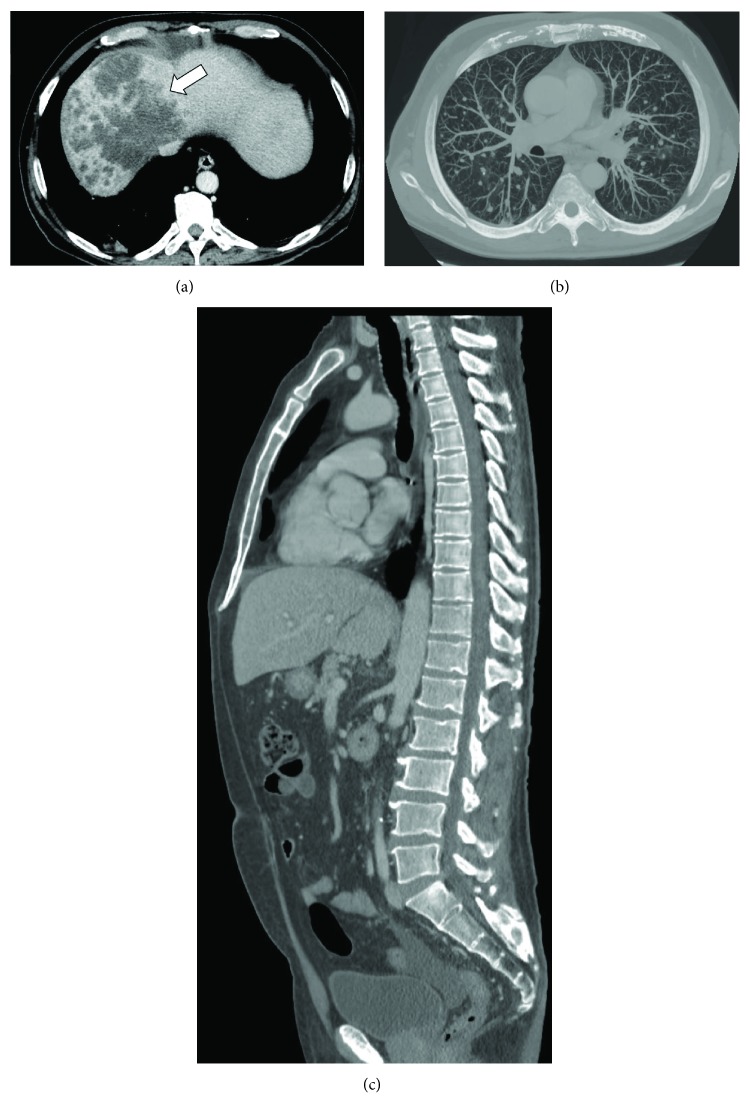
(a) Portal phase CT scan shows large, hypoattenuating mass (arrows) in the right hepatic lobe, consistent with mass-forming peripheral cholangiocarcinoma (arrow). (b) Maximum intensity projection reconstruction of axial CT scan of the chest demonstrates multiple bilateral lung nodules consistent with metastasis. (c) Sagittal CT reconstruction of the spine showed no bony lesions.

**Figure 2:**
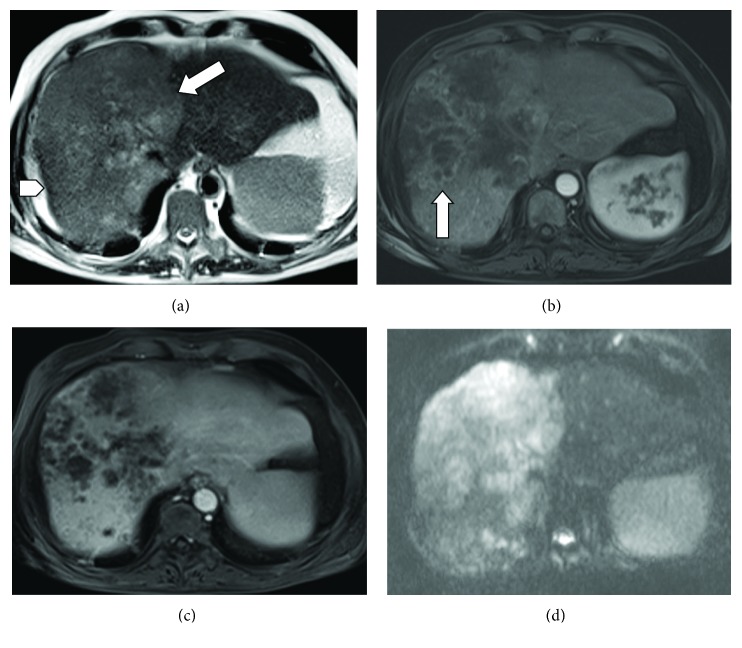
(a) Axial T2-weighted magnetic resonance (MR) image shows a hyperintense mass located in the right hepatic lobe (arrow). There is mild parenchymal atrophy with minimal capsular retraction peripheral to the posterior aspect of the lesion (arrowhead). T1-weighted MR images show irregular, ragged rim enhancement (arrow in (b)) with gradual centripetal enhancement in the portal phase (c). (d) Diffusion weighted image demonstrates high cellularity.

Whole body multidetector computed tomography (MDCT) scan showed a large hypoattenuating hepatic mass in the right lobe (maximum axial dimension 13 cm) characterized by heterogeneous peripheral enhancement, associated with secondary lesions located in the spleen and lung (more than 30 nodules); no evidence of bone metastasis was found (Figure 1). The following magnetic resonance imaging, performed to confirm the diagnostic hypothesis from the previous CT study, showed typical findings of peripheral CHCC (Figure 2). Alpha-phetoprotein was 247.8 ng/mL (normal value < 9 ng/mL), gastrointestinal cancer antigen (GICA) was 132 U/mL (normal value < 37 U/mL), and carcinoembryonic antigen (CEA) was 9.14 ng/mL (normal range 0-5 ng/mL). PTH-rP was markedly high (147 ng/mL; normal range 8.5-20.0 ng/mL).

**Figure 3:**
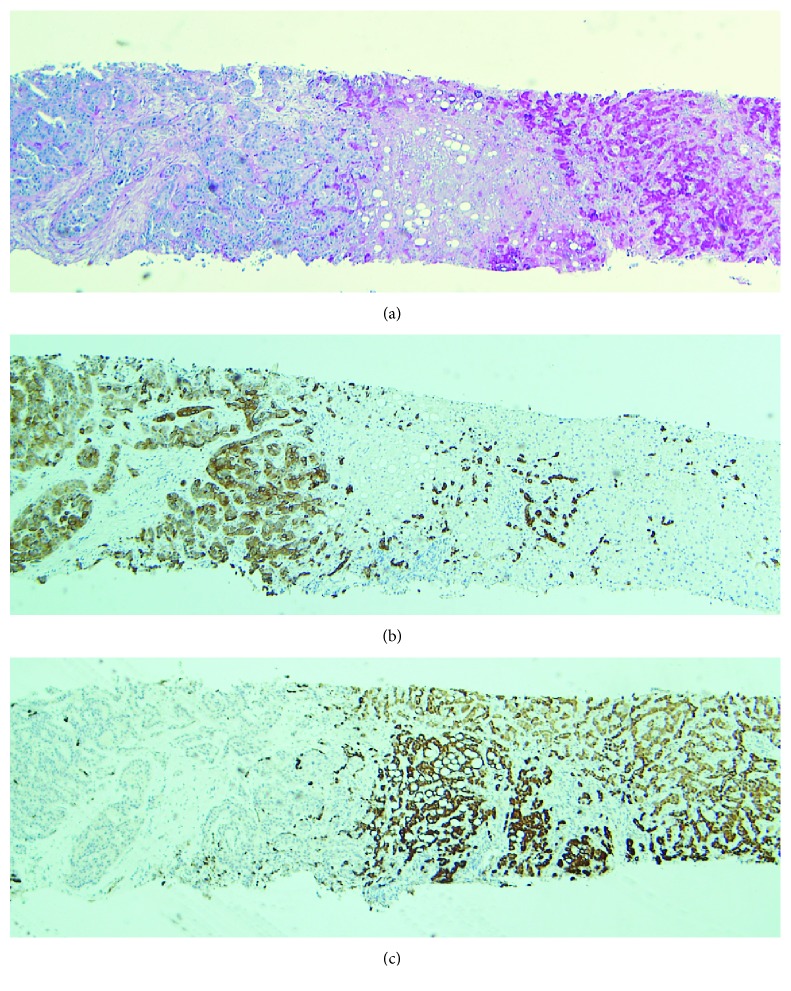
(a) Needle biopsy of the neoplasia showing t solid nests of hepatocarcinoma on the right side with strong cytoplasmic PAS stain and cholangiocarcinoma on the left, with ill formed glands (PAS stain, 1.4x original magnification). (b) Immunoperoxidase staining showed strong CK7 expression on the glandular neoplasia (left side) and negative stain on the hepatocellular carcinoma (right side). (c) Hep-Par1 staining showed a counter-opposite staining pattern (immunoperoxidase stains B and C, 1.4x original magnification).

Percutaneous ultrasound-guided biopsy of the hepatic tumor showed a mixed epithelial neoplasia comprising: (1) trabeculae and solid nests composed of large cells with pleomorphic nuclei and granular cytoplasm intermingled with (2) branching pseudoglandular structures composed of cuboidal/columnar cells with atypical nuclei. Nests and trabeculae showed strong immunohistochemical staining for cytokeratin 8/18 and HepPar1 while pseudoglandular structures stained selectively for cytokeratin 7 and cytokeratin 19 (Figure 3). A diagnosis of “Stage IV CHCC associated to HHM and CIDP” was made.

The general status of patient rapidly worsened, and he became bedridden soon thereafter. After discussion with the patient about the prognosis of his disease, he declined further treatments and arrangements for hospice care were made prior to discharge.

## 3. Discussion

Hypercalcemia of malignancy is typically found in patients with advanced stage cancers and is one of the most life-threatening metabolic disorders. It may result from a marked increase in osteoclastic bone resorption or release of PTH, PTH-rP, or 1,25-dihydroxyvitamin D by the tumor. Acting through a common PTH/PTH-rP receptor, PTH-rP inhibits calcium excretion from the kidney and promotes bone resorption leading to hypercalcemia [3]. As shown in studies investigating the prevalence and prognosis of different paraneoplastic syndromes in HCC, HHM can be found in 4-8% of HCC [4]. HHM has rarely been reported in patients with cholangiocarcinoma (CC) and represents a marker of poor prognosis of the disease [5–9]. CHCC is a rare tumor with poor prognosis, with incidence ranging from 1.0% to 4.7% of all primary hepatic tumors [10].

To the best of our knowledge, this is the third case of CHCC associated with HHM [11, 12] and the first one in a patient with recent diagnosis of CIDP. Involvement of the peripheral nervous system is common in patients with cancer, and any part can be affected [13]. The most frequently reported malignancies associated with CIDP are hematologic (Hodgkin's and non-Hodgkin's lymphomas, Waldenström's macroglobulinemia, chronic myelomonocytic leukemia, hairy cell leukemia, and multiple myeloma). However, CIDP has been described in patients affected by gastrointestinal malignancies (pancreatic, rectosigmoidal, esophageal, and gastric), renal cell carcinoma, lung cancer, seminoma, Kaposi's sarcoma, orbital neurogenic tumor, breast carcinoma, and melanomas, and a possible paraneoplastic pathogenesis of this neurological disease has been suggested [14–33].

It is challenging to determine whether the association of cancer and CIDP is a coincidence or could be explained by a paraneoplastic process. In our patient, CHCC diagnosis was made 3 years after the onset of neurological symptoms and 3 months after the CIDP diagnosis. Since many reported cases identified CIDP prior to the diagnosis of cancer, we supposed that CIDP could represent a paraneoplastic manifestation of CHCC. Although no association between CIDP and CHCC has been previously described, few cases of CIDP associated with HCC [34–36] have been described. Interestingly, one case of CIDP associated with CC has been reported [20], and in that case, cancer was diagnosed about 8 months after the onset of neurological symptoms and 5 months after the CIDP diagnosis.

In conclusion, we report the first case of CHCC associated with HHM and CIDP. Given our case and the other ones reported in the literature, primary hepatic tumors such as HCC, CC, and CHCC should always be included in the differential diagnosis of hypercalcemia of malignancy. Furthermore, in patients with CIDP and elevated aminotransferases, we suggest ordering a full panel of liver function tests, including ALP, yGT, and total bilirubin, looking for a primary hepatic tumor and considering that CIDP associated with primary biliary cirrhosis has been described as well [37]. On the other hand, we suggest that CIDP should be ruled out in patients known to have a primary hepatic tumor presenting with a compatible clinical picture suggesting a demyelinating polyneuropathy.
